# “I live my own life”: relationship between will to live and wish to die in nursing home residents and their opinions about actions to promote their will to live

**DOI:** 10.1186/s12904-026-02238-2

**Published:** 2026-07-25

**Authors:** Samira Ruedin, Laura Jones, Eve Rubli Truchard, Gian D. Borasio, Ralf J. Jox, Marc-Antoine Bornet, Mathieu Bernard

**Affiliations:** 1 Palliative and Supportive Care Service and Service of Geriatric Medicine and Geriatric Rehabilitation, Chair of Geriatric Palliative Care, Lausanne University Hospital and University of Lausanne, Av. Pierre-Decker 5, Lausanne, CH-1011 Switzerland; 2 Service of Geriatric Medicine and Geriatric Rehabilitation, Lausanne University Hospital and University of Lausanne, Lausanne, Switzerland; 3 Palliative and Supportive Care Service, Chair of Palliative Psychology, Lausanne University Hospital and University of Lausanne, Lausanne, Switzerland; 4 Institute of Humanities in Medicine, Lausanne University Hospital and University of Lausanne, Lausanne, Switzerland

**Keywords:** Will to live, Wish to die, Long-term care facilities, Nursing homes, Geriatric palliative care, Intervention, Thematic analysis

## Abstract

**Background:**

The will to live (WTL) is a crucial marker of nursing home residents’ subjective psychological state. Previous studies have indicated that nursing home residents express a relatively strong WTL, and the link with their wish to die (WTD) is ambiguous. There is a lack of literature exploring nursing home residents’ experiences of the co-existence of WTL and WTD and whether and how residents want their WTL to be enhanced. This study aimed to explore (i) the link between WTL and WTD in nursing home residents, (ii) their opinions about support to improve their WTL, and (iii) their suggestions for interventions to achieve this goal.

**Method:**

Participants were nursing home residents aged 70 + living in a nursing home for ≥ 1 month, who maintained decision making capacity. Intensity of WTL and WTD was measured with a single-item numerical rating scale (0 to 10). Semi-structured interviews were conducted to explore residents’ opinions about WTL interventions.

**Results:**

One hundred and nineteen residents participated in the study. Results showed a significant negative association between WTL and WTD (*r*=-.58, *p*=.001), and insights into this relationship are provided through the analysis of qualitative data. 49% expressed that they did not want interventions to support their WTL; 39% were favorable to interventions, and 9% had reservations about accepting such interventions. Regardless of their desire for intervention, most participants provided suggestions regarding the mechanisms through which such interventions could increase WTL: (i) increasing quality human contact, (ii) reducing pain and disability, (iii) facilitating personalized activities, (iv) promoting adaptive institutions with caring and trained staff, (v) promoting opportunities to contribute to others and society and (vi) strengthening their own attitude and levers.

**Conclusion:**

Overall, this study provides a better understanding of the relationship between WTL and WTD, their coexistence, and of the subjective needs and concerns in older nursing home residents, thus providing insights into ways to improve their care and, where desired, their WTL.

**Supplementary Information:**

The online version contains supplementary material available at https://doi.org/10.1186/s12904-026-02238-2.

## Key points

• Will to live and wish to die may co-exist in nursing home residents.

• This co-existence highlights the need to explore both expressions of wish to die and will to live simultaneously.

• Acceptability of interventions to promote WTL is variable. Such interventions could focus on personalized activities and improving quality of connection with others.

• The importance of quality of connection with health professionals highlights the need to ensure working conditions which promote professional well-being so that they have the necessary resources to connect with residents.

Why does this Paper matter?

This study explores the link between the WTL and the WTD in nursing homes and presents the opinion of residents themselves on the relevance of an intervention on the WTL.

**Supplementary Information:**

The online version contains supplementary material available at https://doi.org/10.1186/s12904-026-02238-2.

## Background

Nursing homes are the second most common place of death in Switzerland, after hospitals [1]. In 2022, 5.3% of the Swiss general population and 15% of the population over 80 years of age, resided in nursing homes, 66% of whom were women [2]. The country ranks among those with the highest proportion of elderly people receiving in nursing homes and receiving long term care [3]. People entering nursing homes are in general increasingly older and in poorer health, requiring more and more care [4]. In Switzerland, people enter nursing homes at advanced ages (85.8 years on average), predominantly due to highly developed at home care services [5]. People often live for many months or even years in nursing homes, with an average stay was of 1.7 years for men, and 2.6 years for women [6]. Nursing home residents commonly suffer from multiple morbidities, increasing their vulnerability and complexifying their care and management [7]. Ensuring appropriate care requires attentiveness and responsiveness to residents’ needs and wishes for their well-being.

The will to live (WTL), which has been described as “the psychological expression of one’s commitment to life and the desire to continue living, encompassing both instinctual and cognitive components” [8], is a critical indicator of older peoples’ subjective experience and well-being [9]. A recent scoping review showed that among the 25 methods used in more than 100 studies measuring the WTL, the most commonly used were single-item instruments assessing the WTL intensity [10]. To date, the majority of research has focused on the wish to die (WTD), with less attention given to the WTL. Importantly these two constructs are not simply opposites, and can coexist simultaneously within the same individual [10–13].

The coexistence of WTL and WTD in the elderly can be explained by the fact that, on the one hand, attachment to social ties, routines, perception of a fulfilled life and past identity feeds the WTL. On the other hand, fatigue, isolation, loss and a form of weariness with life can nourish a WTD, which can vary in strength and may be passive. Social support and subjective well-being seem to be key factors in this balance [13]. This ambivalence reflects the final stage of Erikson’s theory of psychosocial development, in which individuals oscillate between integrity and regret [14]. This duality illustrates the complexity of ageing and provides evidence of the fact that a WTD should not be systematically pathologized. A study on the WTD of nursing home residents in Switzerland showed that up to 22% of residents expressed a generally passive WTD, meaning they were waiting for death without desire to hasten it [15]. To our knowledge, the relationship between WTL and WTD has not yet been investigated in older people residing in nursing homes.

Our previous study conducted between waves of the COVID-19 pandemic showed that despite a challenging context, the WTL of nursing home residents was generally high (median = 8/10) and stable and was underestimated by caregivers and family members [16]. According to this previous study, principal factors contributing to the WTL were relationships (especially with family and caregivers), living situations (nursing homes as safe places which necessitate a loss of independence), personality factors (e.g., a positive outlook on life/spirituality), engagement in routines (e.g., organized or individual activities), and health status (especially functional health) [16].

To our knowledge, no studies to date have explored interventions aiming to enhance older nursing home residents’ WTL. Previous research examining the WTL has been primarily observational [10]. Our previous research provides valuable insights on WTL factors determinant for development of interventions to strengthen WTL for nursing home residents, however, it is important to first directly question nursing home residents to assess the relevance and form of such interventions.

The objectives of this study were to explore (i) the link between WTL and WTD in nursing home residents, (ii) nursing home residents’ wishes for support to improve their WTL, and (iii) interventions that nursing home residents suggest for strengthening their WTL.

## Methods

### Design and participants

This is a multi-method observational study, which involved the simultaneous collection of multiple forms (quantitative questionnaire and qualitative semi-structured interview) of data to provide a rich exploration of the topic [17]. The study was conducted in eight nursing homes in the French-speaking region of Switzerland. These institutions were home to a total of 441 residents (range: 20–103 per nursing home), were non-profit and located in metropolitan and rural areas. Inclusion criteria are outlined in Table 1.

**Table 1:** Inclusion criteria

Being aged 70 + years
Living in a nursing home for ≥ 1 month
Having sufficient decision-making capacity to give voluntary informed consent
Having a sufficient level of proficiency in French to participate in the interview
Stable enough health state to participate in questionnaire on recruitment days

The data used here represent only part of the data collected initially in this project, which, in addition to studying the concepts of WTL and WTD, aimed to assess the link between WTL and (i) life-sustaining treatments preferences, (ii) depression, spiritual wellbeing, and (iii) the number of hospitalizations and emergency admissions. Additional variables collected in the initial study (e.g., life-sustaining treatment preferences, depression, spiritual well-being, and healthcare utilization) were not included in this manuscript, as they address distinct research questions requiring separate analyses. While the initial study design aimed to explore these dimensions comprehensively, practical constraints, including limited time and available research resources, did not allow for the full analysis of all collected data within the scope of the present work. Therefore, we chose to prioritize analyses centered on WTL, which was the primary construct of interest and is still underexplored in nursing home populations, particularly in relation to its complex and sometimes ambivalent relationship with WTD. The remaining data have not been published elsewhere and may be the subject of future analyses.

### Sample size calculation

A total of 120 participants was calculated based on the number of independent variables to be included in the initially planned multiple regressions analyses including all quantitative data, assuming 15 participants per variable. The interviews were recorded when the participant consented to being audio-recorded, and iterative analysis was conducted in parallel to the data collection period so that audio-recordings were stopped after 37 interviews when it was established that no new insights relevant to the research questions were being gained.

### Procedure

Recruitment and data collection were conducted between August 2022 and March 2023 (one nursing home at a time). Treating physicians in the nursing homes were informed of the study but had no active role in the recruitment or research processes.

Nursing home residents were identified and listed by the nursing staff according to the inclusion criteria in Table 1, and their health care proxies, were informed about the study by a letter from the institutions seven days before the researchers’ visit. The interviewer (SR or MAB) visited each resident who had not previously informed the institution of their refusal to participate, introduced themself as the researcher working on the study at the regional university hospital, explained the study, answered any questions and provided them with written documentation. Residents were then given a few days to decide whether to participate. Residents wishing to participate signed a written consent form and were included in the study.

Interviews were conducted in person with the resident, alone, or, if the resident agreed, in the presence of the roommate or spouse. Interviews were conducted in the resident’s room or a quiet, low-traffic area of the institution (e.g., a lounge or empty cafeteria). Most (107) interviews were conducted by SR, a trained female psychologist (MPsy) with experience in qualitative interview studies. Twelve interviews were conducted by MAB, a male physician (MD) with 7 years of experience in geriatric research and clinical practice, also experienced in qualitative research. The interviewers practiced interviews with other research colleagues before beginning interviews with residents. In addition, weekly meetings were held to discuss the interviews, to ensure the quality of the interview data and to establish the point at which no further information was being gained for the qualitative analyses, and to stop audio-recording (5 interviews were conducted after no new insights were being gained). Both interviewers were researchers at the regional university hospital and reported no bias or assumptions.

The semi-structured interview guide was developed based on the literature and experiences of the research team and was pretested with relatives and collaborators. The interview guide contained introductory ice-breaker questions (life history, professional life) before discussing the WTL. The participant was then asked to define the concept of ‘will to live’ in their own words. If needed, the definition was discussed to reach similar understanding to that given in the literature : “the psychological expression of one’s commitment to life and the desire to continue living, encompassing both instinctual and cognitive components.” [8] If participants’ responses included aspects of drive to live, desire to continue living, or factors that promote or affect their desire to continue living, the interviewer probed further to verify consistency with the definition in the literature. If participants’ responses did not include these aspects or if uncertainty remained, the interviewer guided participants’ understanding based on the definition [8]. The intensity of the WTD and the WTL was measured with a single-item scale (0–10), as recommended in a scoping review [7], and their interaction explored with an open ended question. Concrete actions to improve the WTL of the residents were explored through a self-developed open-ended question about the interventions residents suggested. Subsequently, a self-developed yes-no question, using the residents’ own suggestions as an example if necessary, explored the openness toward interventions supporting the WTL, and reasons for hypothetical acceptance or refusal of such interventions. This interview guide was developed specifically for this study and an English translation of the specific questions is provided in the supplementary materials.

Interview length ranged from 20 min to 3 h (median:40 min). Participants were informed that the interview could be stopped at any time or paused and resumed later. With the participants’ agreement, 37 interviews were audio-recorded for qualitative analysis. Audio-recordings were transcribed verbatim using Aiko software [18], and transcripts were checked for accuracy and corrected by a native French speaker (SR).

Nursing homes were reimbursed for the time it took to organize informing residents and their families of the study and organizational discussions with the researchers (40 CHF per participant). Residents who participated in the study were thanked for their participation with a box of chocolate.

### Analysis

Bivariate Pearson correlations were conducted to investigate the association between WTL and WTD. In accordance with the exploratory approach of our study, we set a significance level at *p* = .05. The calculation was performed by using SPSS IBM software 29.0 [19]. 

**Fig. 1:**
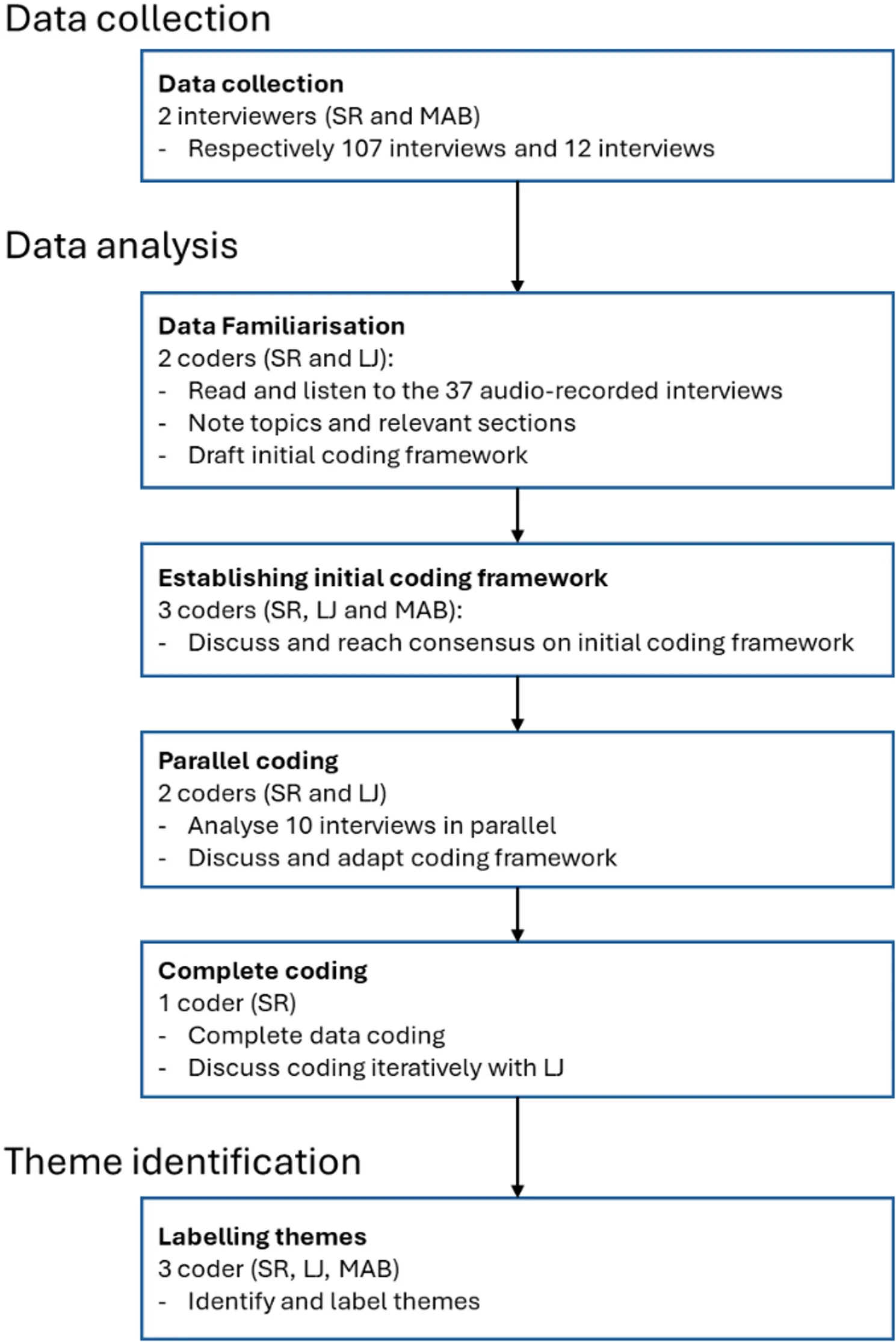
Flow chart of task distribution

The participants’ responses to questions about the co-existence of WTL and WTD, their openness to interventions to improve their WTL and their suggestions for such interventions were analysed thematically. The research questions determined the interview questions, however the participants’ responses were analysed inductively at a semantic level [20]. Two coders (SR, LJ) familiarized themselves with the whole of the data set by reading transcripts, listening to audio-recordings and correcting transcripts, and identified important aspects of the data. Three coders (MAB, SR, LJ) established an initial coding framework, and SR and LJ coded ten transcripts in parallel, meeting every two transcripts to discuss and adapt the coding framework. SR then coded the remaining 27 transcripts, while meeting with LJ iteratively to discuss coding. The steps of the analysis are provided in a flow chart in Fig. 1. Analyses were managed using MAXQDA 2022 [21]. Interviews and analyses were conducted in French, and the extracts presented in this article were translated from French into English and checked for accuracy by a native English speaker.

## Results

**Fig. 2:**
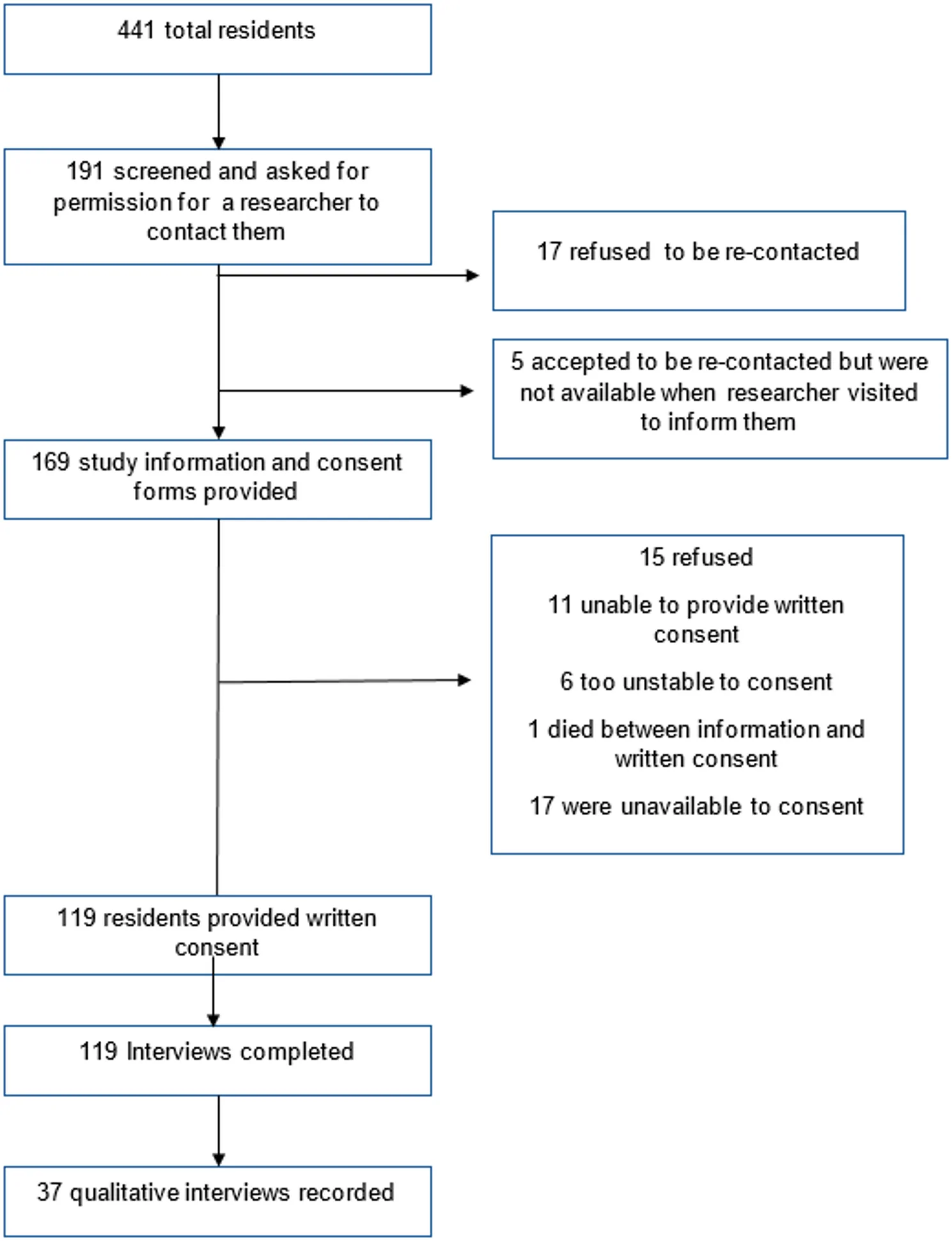
Flow diagram of participants

Of the 441 total residents, 191 were screened as eligible by the nursing staff but 17 refused to see the investigator and five residents were not included because the target number of interviews had been reached and they were not present or available during the times that the researcher was informing residents about the study. Anecdotally, we suspect that this was a form of passive refusal and social desirability in not wanting to actively refuse, so they were not followed up in the interest of not trying to coerce them into hearing about the study. From the 169 residents who were informed, 119 consented and completed the interview (15 refused, 11 were excluded because they couldn’t provide written consent, 6 were finally too unstable to consent, one resident died between information and consent, 17 were unavailable at the time of the consent and interview, probably for the same reasons as already mentioned), as shown in Fig. 2.

### Demographics

Demographic information for participants is provide in Table 2, below.

**Table 2:** Participants characteristics

*All participants characteristics (* *n* * = 119)*		
Age	Mean (M) = 88.1 years, Standard Deviation (SD) = 6.9 years (Range 68–101)	
Gender	Female: 92, Male: 27	
Time in nursing home	Median = 670 days (1.8 years) SD = 916.0, Range: 56-4625 days	
Daily care time	M = 152.7 min, SD = 49.6	
Education	Primary/Compulsory or less Secondary Tertiary	32% 54% 14%
*Qualitative interviews participants characteristics*		
Age	M = 87.7 years, Standard Deviation = 7.6 years (Range 69–101)	
Gender	Female: 25, Male: 12	
Time in nursing home	Median = 875 days (2.4 years), SD = 1019.7, Range 91-4625 days	
Daily care time	M = 152.6 min, SD = 42.1	
Education	Primary/Compulsory or less Secondary Tertiary	27% 57% 16%

### Association between WTL and WTD

**Fig. 3:**
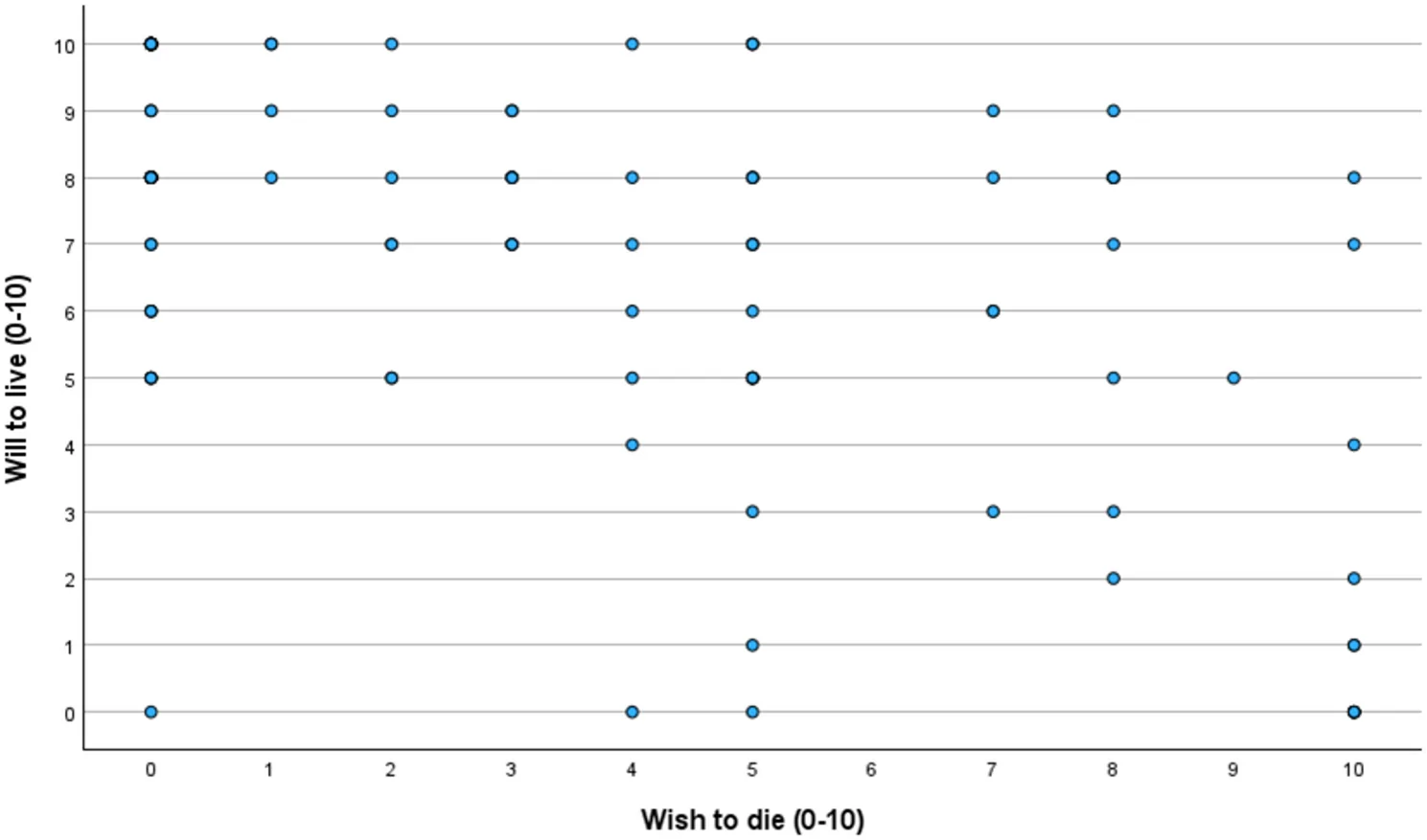
Scatter plot illustrating the relationship between the WTL and the WTD (*n* = 119)

Participants’ mean WTL was 7.0 (SD = 2.9), and their mean WTD was 3.3 (SD = 3.5). The results of the Pearson correlation showed a significant negative association between WTL and WTD (*n* = 117, *r* = − .58, *p* = .001). Although the scatter plot analysis clearly showed this negative association for the majority of our participants, a noteworthy number (11/117) of residents reported moderate to high scores in both WTL and WTD (both ≥ 4, see Fig. 3).

When asked to discuss the links and potential co-existence between wish to die and will to live, participants who expressed a belief that the two could not co-exist typically decribed the two concepts as polar opposites:

“*It’s very polarized. The wish to die is an unknown. So I’m always at the other extreme*” [Participant no. 143, ♂, age 90].

Or, they discussed the states of being as fluctuating and linked to circumstance

*“ So*,* when things are really bad*,* I want to die. And when things are good*,* like today*,* I want to live. That’s how it is*.” [Participant no. 136, ♀, age 89].

Some described the increase of the wish to die as a normal occurrence at a certain age and stage in life :

“*I haven’t really thought about it that much. We all have to die eventually*,* so… But it’s true that it comes with age. You’ll realize that later on. I don’t know if it’s a desire*,* but well*,* it’s a state of mind. [INT: It’s a state of mind rather than a desire? ] Yes. And when you’re old*,* or even before that*,* you don’t do anything*,* you just stay quiet.”* [Participant no. 115, ♀, age 88].

Several also discussed the importance of temprament in these states of being:

“*I want to live. I don’t want to die yet. I’ve never wanted to die. Even at 20*,* with tuberculosis*,* I didn’t want to die. I’m clinging to this world*.” [Participant no. 136, ♀, age 89].

For the people who did believe in the co-existence of wish to die and will to live, they described an acceptation of death coming, however they also have a will to continue living:

“ *So*,* when I don’t think about my son*,* the wish to die is stronger. But when I think about my son*,* it gives me a reason to live*.*”* [Participant no. 4, ♀, age 76].

Furthermore, some participants described the co-existence of a lack of will to live and the lack or slow emergence of wish to die:

” *Ah! (inaudible) The will to live is no longer there. The wish to die creeps in slowly.”* [Participant no. 17, ♂, age 92].

### Willingness for interventions to improve the WTL

Participants’ openness to interventions to improve their WTL was classified into three categories: residents not desiring an intervention, residents desiring an intervention, and residents expressing a willingness for an intervention with some reservation or under certain conditions. Three participants (2%) did not answer this question. Here we describe the reasons given in the qualitative data for these responses in the quantitative data.

#### No desire for an intervention

Among the (49% of) residents who expressed not wanting an intervention to promote their WTL, their reasons included satisfaction with their current WTL level and factors supporting their WTL:

*“There’s nothing more we can do*,* we’ve got everything we need. I think I’ve got everything I need*.” [Participant no. 99, ♀, age 94].

Some participants expressed no longer seeking to improve their WTL at this stage of their life:

*“I think that if someone wants to die*,* you have to let them die. That’s what I think. Because if it’s with reluctance that… you know*,* an effort to be made… I don’t know. Especially if you’re close to death. I’m not saying*,* if it’s someone young*,* that’s another thing. But when you’ve lived your life… I think to myself*,* I’m 93*,* so that’s enough now.”* [Participant no. 99, ♀, age 94].

Finally, some participants expressed that the WTL was too personal to be influenced by external interventions:

*“I think it’s quite personal. If you need someone to give you the will to live*,* then you don’t need it anymore. To want to live*,* it has to come from the heart.”* [Participant no. 113, ♀, age 91].

#### Desire for an intervention

Conversely, 51 participants (39.2%) expressed openness toward an intervention to improve their WTL, with some being very enthusiastic about such a possibility:

*“Yes*,* it’s something that would certainly be positive. That’s for sure.”* [Participant no. 143, ♂, age 89].

A few participants specified that this is the role of the family:

*“Ah*,* but those who want to live*,* what they expect is from their family. Of the outsiders… First of all*,* there’s the question of age*,* which is different. I remember old things that people who come from the outside wouldn’t know.*” [Participant no. 53, ♀, age 87].

#### Desire for an intervention under certain circumstances

Twelve participants (9.2%) stipulated certain conditions for accepting interventions to improve their WTL. For instance, some stated that support for improving their WTL was appreciated, provided they did have too high expectations and as long their health status remained sufficient, but made no sense if the quality and duration of their remaining life were too limited:

“*Of course*,* health*,* as long as I still have a bit of health… but if it’s to hang around*,* I’d rather leave*.” [Participant no. 58, ♀, age 92].

#### Barriers to providing interventions

Amongst both participants who were open to WTL interventions, and those who were not, some discussed the potential barriers to the feasibility of providing WTL interventions. These barriers included health professional time, organizational factors associated with large numbers of people living together and needing care and resources necessary for this within the institution, and the difficulty of personalizing such interventions while also ensuring that multiple people could participate and a harmonious cohabitation:

*“Yes*,* but what would you do? ”* [Participant no. 86, ♂, age 83]

“*No*,* because… I don’t expect anything more here. I know it’s not possible*,* it’s pointless*.*”* [Participant no. 79, ♂, age 89].

### Concrete interventions suggested by the residents

Regardless of whether they wanted an intervention to improve their WTL, most participants provided insights into what could improve their WTL. When residents mentioned concrete interventions, their suggestions varied and were specific to their interests. Six domains were identified: (i) increasing quality human contact, (ii) reducing pain and disability, (iii) facilitating personalized activities, (iv) promoting adaptive institutions with caring and trained staff, (v) promoting opportunities to contribute to others and society and (vi) strengthening residents’ own attitude and levers (Table 3).

**Table 3:** Summary of themes and sub-themes for types of interventions

Increasing quality human contact	Increased contact with loved ones
	Having someone to have deep conversation and deepest feelings with
	Improving connection in the nursing home
	Feeling that someone cares
	Knowing that their loved ones are well
Reducing pain and disability	Reducing pain and discomfort
	Reducing medication
	Better functional health and autonomy
	Loss of ability to partake in past activities due to disability
	Compromise between desires and current possibilities
Facilitating personalized activities	Creative and cultural activities
	Going out of the nursing home/Spending time outside
	Group activities/sharing/bonding
	Participating in daily routines
	Staying occupied and engaged
	Physical activities
Promoting adaptative institutions with caring and trained staff	Available and caring staff
	Adaptive institutions
	Regroup people with similar levels of dependance
Promoting opportunities to contribute to others and society	Being present for loved ones
	Feeling useful
	Not feeling like a burden
	Concern for the future of others/the world
	Providing a legacy
Strengthening resident’s own attitude/levers	Reminiscing about the past
	Being positive/open
	Faith

#### Increasing quality human contact

Increasing the frequency and quality of meaningful contacts (depth of discussions or connection) were frequently mentioned as levers for action that would increase residents’ WTL, whether through in-person interactions with their loved ones or with people in the nursing home, or through the indirect support that their loved ones provide.

##### Increased contact with loved ones

Many residents mentioned contact with their loved ones as a key to their WTL but generally expressed their understanding that their loved ones did their best, and had their own lives to live:

*“Maybe to have visits a little bit more often. They all come. My daughter*,* she works. She said she would come say hello.”* [Participant no. 79, ♂, age 89].

##### Having someone to have deep conversation and share deepest thoughts with

Nevertheless, residents more frequently expressed desiring someone with whom to have deeper discussions, talk about their concerns and deepest feelings, and share their thoughts to improve their WTL:

*“Above all*,* I need someone I can confide in.”* [Participant no. 147, ♀, age 91].

##### Improving connections in the nursing home

Participants also expressed a need for more meaningful contact within the nursing home, sometimes with other residents but mainly with staff, predominantly nursing staff but also with the other personnel in the nursing home:

*“Management doesn’t come to us easily. Well*,* they come to certain people. I don’t know […] right now they just pass us by. Sure*,* they always ask if we’re okay*,* but it’s just a quick question. (INT: There’s no real connection*,* is that what you’re saying? ) No*,* there’s no connection at all.”* [Participant no. 25, ♀, age 75].

##### Feeling that someone cares

Without necessarily needing to physically interact with their loved ones, whom they believe have busy lives, some residents explain that simply knowing that someone cares about them, that someone inquires about them with relatives, or that their relatives are relieved to know that they are well, has a positive impact.

*“If someone asks how I am*,* it means they are interested in me to some extent.”* [Participant no. 48, ♀, age 102].

##### Knowing their loved ones are well

In the same manner, some explained that simply knowing that their loved ones are well, safe, fulfilled, and thriving on different levels promotes their WTL:

*“Simply that my children are well. That’s all I ask for.”* [Participant no. 100, ♀, age 87].

#### Reducing pain and disability

The most frequently cited elements of WTL were often related to residents’ health, mainly through reducing pain and discomfort, reducing medication, improving functional health and autonomy. Although participants often considered themselves fortunate compared to their peers regarding their health status and expressed awareness that their wishes regarding their health are unrealistic, they often mention the loss of possibilities entailed by their health status, and sometimes expressed that compromises would be acceptable.

##### Reducing pain and discomfort

Although they often considered themselves fortunate compared to their peers regarding their health status, reducing pain and discomfort was frequently expressed as levers for improving the WTL, although often perceived as unrealistic:

*“To be a bit better on the health level. I’m not asking to be fine. That’s over. As Mr. (Doctor) says: ‘I can’t take 5 years off of your life’. Because*,* at 80*,* I was still fine. But to be less dependent … The pain too*,* it’s very distressing to always be in pain.”* [Participant no. 113, ♀, age 91].

##### Reducing medication

A few participants noted that the increased number of medications they had to take was a burden, and that reducing these could improve their WTL:

*“All these medications*,* it’s not… It’s a lot. And then it helps with something*,* but then he said it’s not good for others.”* [Participant no. 25, ♀, age 75].

##### Better functional health and autonomy

Many participants mentioned aspects of their functional health such as vision, hearing, mobility, manual dexterity, strength, balance, or cognitive abilities such as memory, as disabilities limiting their daily activities and autonomy, and affecting their will to live:

*“Again last night*,* because I have a commode chair at night that they put there*,* and when I got up last night*,* I felt dizzy. I was standing up*,* and the dizziness threw me backwards. Luckily*,* there was the bed*,* but I hit it hard. So now both my elbows hurt. I wish I could do things without hurting myself every time*.*”* [Participant no. 113, ♀, age 91].

##### Loss of ability to partake in past activities due to disability

Functional improvements were discussed as linked to activities residents enjoyed in the past but were no longer able to participate in. They were also linked to the possibilities of new activities and experiences and of leaving the nursing home to return home:

*“The ability for my wife to walk*,* to get back. That would make a big difference. [INT: Would that make things better for you too? ] Yes. We have a house*,* but we can’t go there because she wouldn’t even be able to get in because there are a few steps. You see*,* there are things like that. Then*,* of course*,* there are the eyes and ears*,* to be able to connect with what’s going on.”* [Participant no. 143, ♂, age 89].

##### Compromise between desires and current possibilities

Some residents discussed the possibility of alternative access to an activity as a compromise, such as listening to an audiobook when poor eyesight rendered reading impossible. However, others indicated that compromises would be unsatisfying (e.g., using a wheelchair):

*“Yes*,* what would be nice is an opportunity to improve my balance so I can get around. And then*,* who knows*,* maybe go on another cruise*,* or take another trip; things like that. But I want to be free to do my own thing*,* not be dependent on anyone. I’ve been on several trips and cruises. I really enjoyed it*,* but I can’t see myself doing it in a wheelchair. I have a certain sense of my limits*,* and I don’t want to depend on anyone. If I do it again*,* it means I’ll be free to do what I want.”* [Participant no. 85, ♂, age 85].

#### Facilitating personalized activities

Residents mentioned various activities as ways to improve their WTL that depended on personalities and personal interests.

##### Creative and cultural activities

They mentioned creative activities, including painting, writing, crafting, knitting, participating in a mechanical workshop, but also cultural activities, such as intellectual games; attending the cinema, museums, concerts, or exhibitions.

*“… to see the military bands again. I watch that on TV*,* they call it tattoos […] at [Place]. They put them on the newspaper*,* I watch them all […] [INT: And then is there something else you could perhaps do yourself to improve your will to live? ] I thought I could have an instrument and try to blow.”* [Participant no. 16, ♂, age 93].

##### Going out of the nursing home/spending time outside

Participants also often expressed the need to spend time outside; traveling and discovering other countries; and returning to places dear to their hearts, such as the mountains or the lake. Leaving the nursing home more often, even if only walking in the garden, was a frequently cited element:

*“Walking*,* strolls*,* the soil*,* the mountains…* [Participant no. 76, ♀, age 76].

##### Group activities/sharing/bonding

Several participants also mentioned activities related to sharing or bonding, seeing friends:

“*To have activities with groups or with someone*,* I don’t know*,* from outside*.” [Participant no. 114, ♀, age 94].

##### Participating in daily routines

The need to feel useful, helpful, and service-oriented was described as beneficial by several residents, both for connection with others and self-esteem:

“*I like to help clearing the tables*,* cleaning the tables*,* cleaning the placemats”* [Participant no. 127, ♀, age 82].

##### Staying occupied and engaged

Boredom and the need to simply pass the time were also mentioned by several residents, without necessarily highlighting a particular activity that would be meaningful to them in itself:

(Having a computer) “*Let’s say it would improve my empty days. [INT: It would give you something to do? ] It would fill a certain void in my days*.” [Participant no. 85, ♂, age 85].

##### Physical activities

Finally, some suggestions were linked to physical needs and activities, such as exercising with a home trainer or the possibility of sexual activity:

*“Making love from time to time […][Int: Anything else that could improve your zest for life? ] Being able to go running. I miss that. […] I’d like to have an exercise bike*,* a bike to pedal.* [Participant no. 47, ♂, age 92]

#### Promoting adaptive institutions with caring and skilled staff

The organization and resources specific to the nursing home were also important points raised by residents, whether in regard to the availability of staff, flexibility within the institution, and organizational factors within the institution.

##### Available and caring staff

Increased availability and understanding from the staff were suggested as a lever to improve WTL. Although generally aware that they were doing their best, the residents felt that nursing staff lacked time and wished for more interpersonal skills and availability as well as fewer task-oriented conversations with more in-depth interactions:

*“More staff. Or make them more aware of what they do and how they are [interpersonally]. […] And there should be more staff*,* because that would be… […]I’m not aware of the training given to staff*,* and I’m not talking about nurses. But we could put nurses in. The employees*,* the nursing staff*,* for basic care*,* I feel their stress […] And it blocks the relationship […] They’ve become used to … A quarter of an hour*,* or ten minutes*,* for a shower. They’re now formatted to act like that*,* and it’s going to be difficult to correct.* [Participant no. 4, ♀, age 76]

##### Adaptive institution

More flexibility and resources in the nursing home were suggested by some residents, for example, regarding wake-up times, mealtimes, and meal choices:

*“Yes. Now it’s not the case because I have to limit myself. […] We have a meal plan here. Sometimes it’s good*,* sometimes it’s not so good. I work with it*,* that’s for sure. [INT: Would you like to be able to choose according to your mood? ] Yes*,* depending on what I want.”* [Participant no. 86, ♂, age 83].

##### Regroup people with similar levels of dependance

Some residents identified being affected by the daily presence of people who were much more impaired than they were and suggested specialized institutions for people who retained their cognitive abilities but needed daily living assistance:

*“There are people who don’t respond anymore*,* who don’t know how to eat by themselves*,* who… Me*,* I’ve got my mind. That’s what’s hard. I’ve got my mind; I see everything*,* I hear everything… At 74*,* thank goodness. But… They should make nursing homes for people who’ve had a stroke and who aren’t completely… there you go.”* [Participant no. 25, ♀, age 75].

#### Promoting opportunities to contribute to others and society

Many residents expressed that a sentiment that their existence still contributes positively and that they can still pass something on to their loved ones (whether financially, materially, or emotionally) is instrumental in promoting their WTL:

##### Being present for loved ones

Being present for their children and grandchildren, particularly during important life events, is also a factor mentioned by residents as giving them a renewed WTL.

“*Not anymore… I don’t mind leaving. But now… It’s true that there will be… She’s the last of my granddaughters that I thought would have…* [a baby]” [Participant no. 38, ♀, age 94].

##### Feeling useful

Contributing, even in a small way, is also a way for some participants to feel useful and thereby improve their WTL:

“*Not just any way*,* of course. But even now*,* at 84*,* I still want to live. I do things for others too.*” [Participant no. 15, ♂, age 85].

##### Not feeling like a burden

Some participants specifically mention their desire not to be a burden on their loved ones, whether financially or emotionally.

“*It’s when I see my family*,* when everyone is well*,* everyone is happy*,* they’re happy to see me. And they still do their best to take care of me. But if I tell them that I’m not okay*,* that it’s not necessary*,* that they should take care of themselves first. Then they accept it. And then I don’t know where I am*,* but at least I’m not in their way. Because they’re all younger*,* they need to live their lives*,* and I don’t want to be a barrier to that. So I give them complete freedom. […] so that I’m not a burden. So*,* they accept me as I am*,* but I don’t stop them from… If there’s something*,* they come first.”* [Participant no. 8, ♀, age 104].

##### Concern for the future of others/ the planet

Some residents also express concerns about the future of their loved ones and the environment/world they would leave behind. They believe that peace of mind regarding these concerns would also be a driving force behind their WTL.

“*And then that nature does not deteriorate*,* that we can continue to enjoy it.”* [Participant no. 82, ♀, age 93].

##### Providing a legacy

Whether through the inheritance they have set aside for their children over the years or through a desire to pass on values or memories, participants expressed a desire to protect and pass on a legacy. Some expressed concern about the financial impact of their situation. These residents stated that they would appreciate benefitting from more financial resources to avoid feeling like a burden to their family and to enable them to leave behind an inheritance:

*“I must say that… financially*,* it plays a big role*,* doesn’t it? Because… we shouldn’t kid ourselves. A day in hospital or a day here-”* (referring to hospital costs) [Participant no. 38, ♀, age 94].

#### Strengthening residents’ own attitude/levers

Some residents also mention intrinsic elements within their control, related to their own attitudes.

##### Reminiscing about the past

Although for some, thinking back on the past is seen as having a negative impact, revisiting fond memories was reported as a lever which can improve their WTL:

“*You see*,* it would mean looking back at the past*,* at professional life and working life in different contexts.”* [Participant no. 15, ♂, age 85].

##### Be positive / open

A positive attitude towards oneself, working on oneself and encouraging oneself is also mentioned by some participants as a means by which they can improve their own WTL.

“*Ah*,* but that’s personal. You can tell yourself*,* “You’ll see*,* it’ll be okay*,* you’re not alone*,* be brave*,*” etc. But we can do that ourselves. Because it’s all very well for the person opposite you to say*,* “You’ll see*,* it’ll be okay*,*” etc.*,* but they’re not in our shoes*,* are they?”* [Participant no. 136, ♀, age 89].

##### Faith

For some, faith is also an important resource that allows them to work on their own WTL.

“*Well*,* yes*,* I can say that. That’s how it is. I think we have to accept what happens to us*,* if possible. We have to think carefully about what we should and shouldn’t do*.*”* [Participant no. 82, ♀, age 93].

## Discussion

To our knowledge, this study is the first to explore the link between WTD and WTL in nursing home residents. Regarding the quantitative part, the high average score of the WTL and the low average score of the WTD should encourage healthcare professionals to keep in mind that the willingness to live is still present and needs to be assessed when caring for older nursing home residents, even if our participants are not representative of all residents (see limitations). The WTL of this population is slightly lower (7/10 in this study) compared to previous research (4.05/5) in previous research [22], however this difference (1.1/10 points) is likely to be clinically insignificant. The WTD was measured as a continuous variable and the mean score was relatively low (3.4/10); previous research in a Swiss hospitalized geriatric population established the prevalence of WTD at 14.9% [23]. Due to differences in measurement tools, however, it is difficult to compare these results. Further research could examine the differences between WTD and WTL across living settings and health conditions in geriatric populations. In addition, we showed a strong negative correlation (-0.58) between the WTD and the WTL. However, around 10% of the residents reported a moderate to high score in both dimensions. Some of the residents have a strong WTD, but with an ambivalence, because at the same time they want to continue their lives. This proportion of patients is not negligible from a clinical point of view and illustrates the need to look beyond the overall correlation score alone to draw conclusions that are meaningful for clinical management. This ambivalence had already been demonstrated in several studies conducted with older adults and in palliative care or end-of-life settings [13, 24–26]. Our qualitative exploration of the links between WTL and WTD provides a better understanding of the ambiguity emerging for some residents.

The qualitative analyses about openness to interventions indicate that, while approximately half the participants were open to interventions to enhance their WTL, it did not appear to be a primary concern of nursing home residents, who tended to be more pragmatic and focus instead on the pleasure of living and quality of life. Willingness varied between people. Hence, it is important to ask residents directly before acting to improve their WTL to avoid imposing on a dimension they may consider too personal and intimate, thus violating their autonomy.

Residents were initially uncertain about what an intervention could involve and how it could improve their WTL. They often required further explanation before proposing ways to improve their WTL. This pattern and their first reaction to the question suggested they were generally happy with their situation or that this facet of their life was too existential in nature to be influenced by an intervention.

We identified desires and areas for potential improvements to nursing home residents’ WTL. The suggestions for interventions mentioned by the residents were highly variable and specific to each person’s interests, leading to the same conclusion: it is best to ask residents about their values and perspectives to ensure a personalized approach and use resources efficiently. These suggestions included increasing quality human contact, reducing pain and disability, facilitating personalized activities, promoting adaptive institutions with caring and trained staff, and keeping wealth to pass on. They were similar to the themes at the source of the WTL, as we have observed previously [16]. The current study, while confirming the importance of these aspects in residents’ lives, extends previous findings by identifying factors upon which to base WTL interventions in this context.

The broad factors identified in this study are coherent with the domains affecting quality of life of nursing home residents identified in a recent systematic review [27], which is to be expected, since quality of life is an inherent part of the WTL according to Carmel [22]. However, it is noteworthy that spirituality, which was identified in our previous research [16] was not identified by the residents as something that can be worked on, possibly due to its highly individual nature. Other domains of health and autonomy have also been identified in our research, with a tone of resignation to living with a state of health that impacts residents’ autonomy. The concepts of sense of place are also highlighted in our study, when participants, although they often would rather go home, discussed being ‘well’ and having their needs met, however they did not mention a sense of ‘homeliness’ as a factor that could be improved upon in improving their WTL. Finally, Rodriguez-Martinez et al. also identified the domain of relationships, and this is one of the principal levers of WTL identified in our study, thus highlighting its importance in both quality of life and WTL [27]. 

This study also shows nursing home residents’ ability to adapt to their living conditions. However, it also shows the impact of the staff, their workload, and frequent changes, on residents, suggesting that, like satisfaction with care, state of health, risk of accident, and dependency level [28–31], has an influence on residents’ WTL. This highlights the importance of improving nursing home staff working conditions to ameliorate residents’ well-being.

### Implications for clinical practice and research

Although the WTL may be a target for improvement for some people, the variability in acceptability of such an intervention indicated the need to discuss WTL according to each residents’ openness to such discussions and to develop interventions that are adaptable. The WTL is a particularly intimate element relating to personal volition, personality, and meaning in life; thus, it may be ethically questionable to intrude by trying to influence it without first verifying the individual patient’s stance. Generally, the WTL could be better observed and used as a tool or catalyst for a discussion to better understand each individual’s subjectivity, as we have suggested elsewhere [16], or as an indicator of well-being useful in diagnosis and prognosis, as proposed by Carmel [22, 32], rather than as something to be actively influenced. This sets the WTL apart from quality of life, which several residents mentioned as the most important area for improvement. Our high participation acceptance rate indicates that WTL is a topic that nursing home residents are open to discussing; it is however important that people discussing WTL are adequately trained and prepared and that the necessary skills, particularly in terms of active listening and emotion management, are taken into account when determining which professionals are best placed to conduct such discussions.

### Limitations

First, the present analyses are based on a subset of a larger dataset. Although this selection was guided by the specific research objectives focusing on WTL and its relationship with WTD, the exclusion of other collected variables (e.g., depression, spiritual well-being, healthcare utilization, and treatment preferences) limits the comprehensiveness of the analyses. The absence of these variables prevents a more integrative examination of factors that may influence WTL, including psychological, spiritual, and clinical dimensions. As a result, potential confounding or interacting effects cannot be fully accounted for, which may affect the internal validity of the findings. Furthermore, this focused analytical approach may limit the generalizability of the results to broader models of WTL that incorporate these additional dimensions. The findings should therefore be interpreted as a targeted exploration of the relationship between WTL and WTD, and of residents’ perspectives on interventions, rather than as a comprehensive account of all determinants of WTL. Future research should aim to integrate these multiple dimensions to provide a more holistic understanding of the will to live in nursing home residents.

Although the participants shared many personal and intimate elements during the interviews, certain elements may not have been raised because they were too personal to share with a researcher. Moreover, a social desirability bias could not be ruled out, so some participants may have conveyed a more socially acceptable version of their experiences.

Notably, according to the participants’ feedback, the questions of willingness to intervene and suggestions for interventions to improve the WTL were relatively difficult to answer. Since the WTL is a highly abstract and existential concept, it is possible that when establishing concrete elements for interventions, they are aimed at generally improving quality of life.

The question of the desire for interventions to improve the WTL was not concrete, possibly making the answer difficult. However, it followed the suggestions proposed by the residents in chronological order. Indeed, one could imagine that participants would be more open to directly proposed concrete interventions. Nevertheless, the intention here was to assess the extent to which the WTL was too intimate to be voluntarily influenced, and the contrasting results and explanations given by participants demonstrated that this question, while vague, achieved its objective.

Furthermore, although the participation rate was relatively high (67%), those residents who did accept participation are likely to be in better physical and psychological health than the general nursing home population, thus diminishing the generalizability of the results. In addition, the nursing homes which were invited to participate were known to be receptive to research projects, and thus likely to be more invested in institutional improvements. It is therefore possible that they have more favorable organizational practices and that their staff and residents are doing better than average.

## Conclusion

This study provides information about the levels of WTL and WTD in the context of nursing home and provides a better understanding of nursing home residents’ subjective needs and concerns, including the meaning they give to the WTL. It shows how WTL and WTD, although strongly negatively correlated, may nevertheless co-exist within the same individual for a small but non-negligible portion of residents. Our findings confirm that the idea that WTL and WTD are mutually exclusive is outdated. On the contrary, the coexistence, sometimes simultaneous, sometimes fluctuating, of these two attitudes is increasingly better documented, complex, and should be recognized as a legitimate part of the end-of-life experience for some residents. These results highlight the need to explore both expressions of WTD and WTL for a better understanding of residents’ experiences. Importantly, we believe it is essential to go beyond a simple numerical assessment of the intensity of the WTL in a clinical context, by exploring the relevant life domains and sources of WTL. This not only provides a better understanding of the ambiguous relationship that exists for some people between these two concepts but also suggests possible interventions for professional teams by considering the specific characteristics of each resident.

These results also provide an additional foundation for using the WTL as a catalyst for understanding subjectivity, as described by Carmel et al.[22]. The WTL could thus be a useful catalyst for exploring residents’ experiences and preferences rather than an objective care outcome measure to be improved. Nevertheless, this study shows that some people wish to receive support to improve their WTL, and provides clues on how care can be improved to enhance the WTL in such cases.

## Supplementary Information

Supplementary Material 1.

## Data Availability

The datasets generated or analysed during the current study are not publicly available as these data are qualitative and publishing them may provide enough information to identify the participants. Selected extracts have been translated and anonymized for publication here. Data will be made available upon reasonable request from the corresponding author for the purposes of verification.
