# Supplementary material for: “I live my own life”: relationship between will to live and wish to die in nursing home residents and their opinions about actions to promote their will to live
Source: BMC Palliat Care. 2026 Jul 25;25:315. doi: 10.1186/s12904-026-02238-2 (PMC13685087; doi:10.1186/s12904-026-02238-2)
Supplement: Supplementary file 1 [file 12904_2026_2238_MOESM1_ESM.docx]

***Interview : Full form***

- Before getting to the heart of the matter, could you tell me what you did for a living? What training do you have?
- As a researcher, I'm interested in the notion of the “will to live”. Have you ever thought about it? What words come to mind?
- Can you evaluate your will to live, by giving a number between 0 and 10, where 0 corresponds to no WTL and 10 to a complete WTL?
- Let us move on to another topic. Have you ever heard of advance directives? I am a researcher, so it is not my role to complete these directives with you, but for the study, I'm going to ask you a quick question on the subject: if something were to happen to your health, what care would you want?
- If you had to define your treatment preferences on this scale, which box would you choose?

| Maximum care, including resuscitation | Maximum care, excluding resuscitation | Go to hospital or emergency department, but not intensive care, intubation or resuscitation | Receive all possible care in EMS to get well, but do not go to hospital | Aim only for comfort, not to prolong life |
| --- | --- | --- | --- | --- |

*Derived from the Physician Orders for Life-Sustaining Treatment (POLST) Form framework (1-2)*

- **GDS-4 scale** (3)

1. Do you often feel down and sad?
2. Do you feel your life is empty?
3. Are you happy/well most of the time?
4. Do you think your situation is hopeless?

- **FACIT-Sp. 12** (4)

Sp1	I feel peaceful

Sp2	I have a reason for living

Sp3	My life has been productive

Sp4	I have trouble feeling peace of mind

Sp5	I feel a sense of purpose in my life

Sp6	I am able to reach down deep into myself for comfort

Sp7	I feel a sense of harmony within myself

Sp8	My life lacks meaning and purpose

Sp9	I find comfort in my faith or spiritual beliefs

Sp10	I find strength in my faith or spiritual beliefs

Sp11	My illness has strengthened my faith or spiritual beliefs

Sp12	I know that whatever happens with my illness, things will be okay

- **Categories of Attitudes Toward Death Occurrence (CADO)** (5)

I am going to present you a series of possible attitudes toward death.Please let me know which of them best corresponds to your attitude toward death

1. I am not ready for death, and I do not accept it.
2. I am not ready for death, but I accept it.
3. I am ready for death, and I accept it.
4. I am ready for death, I accept it, and I wish death would come.
5. I am considering hastening my death, but I have no specific plan.
6. I am considering hastening my death, and I have a specific plan.

- Can you estimate your wish to die by giving a number between 0 and 10, where 0 corresponds to no wish to die and 10 to a maximum wish to die?
- What concrete things do you think could improve your will to live? Give three suggestions.
- If you think about your daily life, what could improve your will to live?
- Is there perhaps something your loved ones could do to improve your will to live?
- Is there anything here at the nursing home that could improve your will to live? Perhaps in the contacts, activities or environment?
- Is there anything you could do to improve your own WTL?
- We talked about what could improve your WTL, I would now like to know if you would want external support aiming to improve your WTL?
  - Do you think it is the place of your family, the nursing home teams or society in general to improve and act on your will to live? Or is it your own business?
- Who are the people you can really count on when you need help? Please list all the people you can think of.

**References**

1. Bomba, P. A., Kemp, M., & Black, J. S. (2012). POLST: An improvement over traditional advance directives. Cleveland Clinic Journal of Medicine, 79, 457-464. https://doi.org/10.3949/ccjm.79a.11098
2. Hammes, B. J., Rooney, B. L., Gundrum, J. D., Hickman, S. E., & Hager, N. (2012). The POLST program: A retrospective review of the demographics of use and outcomes in one community where advance directives are prevalent. Journal of Palliative Medicine, 15, 77-85. https://doi.org/10.1089/jpm.2011.0178
3. Thomas, P., Hazif-Thomas, C., & Clément, J. P. (2008). [Geriatric depression scale version in 4 items (GDS-4)]. *La Revue de Gériatrie*, 33(12), 733-734. https://doi.org/10.1016/j.revger.2008.06.003
4. FACIT.org. (n.d.). *Functional Assessment of Chronic Illness Therapy - Spiritual Well-Being 12 Item Scale (FACIT-Sp-12)*. Retrieved March 14, 2024, from https://www.facit.org/measures/facit-sp-12
5. Durst, A. V., Spencer, B., Bula, C., et al. (2020). Wish to die in older patients: Development and validation of two assessment instruments. *Journal of the American Geriatrics Society, 68*, 1202-1209.  https://doi.org/10.1111/jgs.16392
